# Mediastinal Thyroid Carcinoma and Graves' Disease: A Rare Presentation

**DOI:** 10.1155/2021/6584616

**Published:** 2021-10-12

**Authors:** Sara Lomelino Pinheiro, Inês Damásio, Ana Figueiredo, Tiago Nunes da Silva, Valeriano Leite

**Affiliations:** ^1^Serviço de Endocrinologia, Instituto Português de Oncologia de Lisboa Francisco Gentil, Rua Professor Lima Basto, 1099-023 Lisboa, Portugal; ^2^Unidade de Investigação em Patobiologia Molecular, Instituto Português de Oncologia de Lisboa Francisco Gentil, Rua Professor Lima Basto, 1099-023 Lisboa, Portugal

## Abstract

**Background:**

Mediastinal thyroid carcinoma is extremely rare, with few cases reported in the literature. *Case Report*. A 73-year-old man presented with weight loss for 6 months. Imaging by computed tomography (CT) documented a large mediastinal mass below the thyroid gland and pulmonary metastases. Neck ultrasound found two spongiform nodules in the right thyroid lobe, and fine-needle aspiration citology (FNAC) of these nodules revealed they are benign. Endobronchial ultrasound-guided needle biopsy of the mediastinal mass was compatible with papillary thyroid cancer. A few weeks later, the patient developed overt hyperthyroidism due to Graves' disease, which was treated with antithyroid drugs. ^99m^Pertechnetate scintigraphy showed increased diffuse uptake in the thyroid parenchyma but the absence of uptake in the paratracheal mass and in the lung nodules. The patient was not considered eligible for surgical intervention or therapy with tyrosine kinase inhibitor due to tracheal and mediastinal vessel invasion and was treated with palliative radiotherapy. Two months later, restaging PET-FDG showed an intense uptake in the right lobe of the thyroid gland, lymph nodes, lungs, bone, muscle, myocardial, kidney, and adrenal gland.

**Conclusion:**

In this case, thyroid carcinoma presented as a mediastinal mass with concurrent hyperthyroidism due to Graves' disease. Although uncommon, the clinicians should be aware of these situations. Obtaining a prompt histological examination of an intrathoracic mass is crucial to ensure an early diagnosis and treatment.

## 1. Introduction

Mediastinal thyroid cancer is extremely rare, with few cases reported in the literature [1–3]. Surgical excision of the malignant lesion, whenever possible, is the treatment of choice [4]. In this clinical case, the distinction between ectopic and metastatic thyroid carcinoma is challenging.

We describe a case of papillary thyroid carcinoma (PTC) presenting as an intrathoracic mass with lung metastases. Besides the unique presentation, this case had an atypical course, with subsequent hyperthyroidism due to Graves' disease and widespread cardiac, muscle, and adrenal involvement.

## 2. Case Presentation

A 73-year-old man presented with a 6-month history of unquantified weight loss. His past medical history included hypertension, type 2 diabetes, and smoking (750 pack-years). There was no record of previous head and neck radiation and no relevant family history. Physical examination showed emaciated appearance. Neck examination revealed a palpable nonadherent nodule in the right lobe of the thyroid gland.

### 2.1. Diagnosis

**Figure 1:**
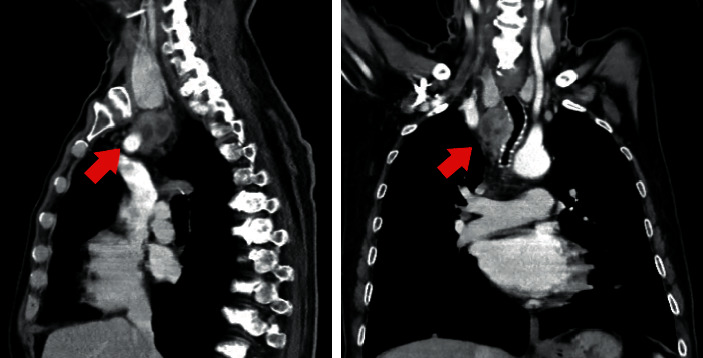
Neck and chest CT. The sagittal image revealed a mediastinal mass (41 × 29 mm) with clear demarcation form the right lobe of the thyroid gland. The coronal image shows the mediastinal mass interposing between the trachea and supra-aortic vessels.

Imaging by computed tomography (CT) documented a large mediastinal mass (41 × 29 mm) inferior to the right lobe of the thyroid gland, interposing between the trachea and supra-aortic vessels, with probable tracheal and mediastinal vessel infiltration (Figure 1). The mass was initially considered a mediastinal lymph node due to its clear demarcation from the adjacent thyroid gland (Figure 1). Chest CT demonstrated multiple pulmonary lesions suggestive of metastases. Endobronchial ultrasound-guided needle biopsy of the mediastinal mass was compatible with PTC (papillary structures with nuclear pseudoinclusions) with thyroglobulin (Tg) in the washout of the needle above 30.000 ng/mL. Tumour cells showed positive staining for Tg, transcription factor 1 (TTF1), and PAX8. There were no cytologic features of undifferentiated tumour in this specimen. Serum Tg was 111 ng/mL (NR: <55). Neck ultrasonography (US) showed two spongiform nodules in the right thyroid lobe with 15 mm and bilateral micronodules. Fine-needle aspiration citology (FNAC) of the two spongiform nodules revealed benign lesions. Upper gastrointestinal endoscopy demonstrated oesophageal compression without significant stenosis or mucosal changes. Bronchoscopy was performed and revealed tracheal infiltration by the tumour. Blood tests confirmed a euthyroid state. The patient was diagnosed with metastatic thyroid cancer.

**Figure 2:**
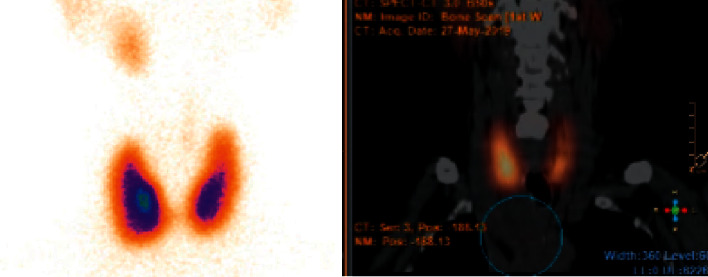
^99m^Pertechnetate scintigraphy showing increased diffuse uptake in the thyroid parenchyma and the absence of uptake in the mediastinal mass.

While awaiting the staging exams, the patient referred palpitations, hand tremors, and diarrhoea. Physical examination showed tachycardia without signs of goiter or thyroid ophthalmopathy. Blood tests identified overt hyperthyroidism due to Graves' disease: TSH <0.02 *μ*UI/mL (NR: 0.30–4.20), free thyroxine (FT4) 3.86 ng/dL (NR: 0.90–1.70), free triiodothyronine (FT3) 9.2  pg/mL (NR: 2.0–4.4), and thyroid-stimulating antibodies (TRABS) 21.9 UI/L (NR: <1.8). ^99m^Pertechnetate scintigraphy showed increased diffuse uptake in the thyroid parenchyma but the absence of uptake in the paratracheal mass and in the lung nodules (Figure 2).

### 2.2. Treatment

The patient was treated with methimazole (MMI) 30 mg and propranolol 60 mg per day, and there was clinical improvement after correction of the thyroid hyperfunction.

Due to the extension of tracheal invasion and disseminated disease, the patient was not eligible for surgical intervention. Therapy with TKI was also not considered because of the patient's general condition and mediastinal vessels infiltration, given the risk of potentially fatal bleeding in case of significant tumour reduction. The mediastinal mass was treated with 20 gray of palliative radiotherapy.

### 2.3. Outcome

**Figure 3:**
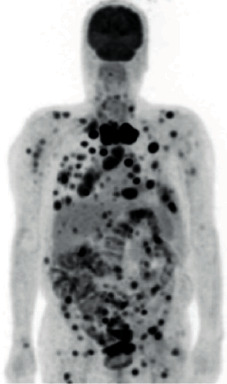
FDG-PET revealed multiple lesions with intense FDG uptake due to malignant disease (thyroid, mediastinal lymph nodes, lungs, bone, muscle, heart, liver, and adrenal).

Two months after completion of radiation treatment, the patient described severe pain in the chest. Restaging fluorodeoxyglucose- (FDG-) positron emission tomography (PET) showed intense uptake in the right lobe of the thyroid gland, lymph nodes, lungs, pleura, bone, muscle, myocardium, liver, kidney, and adrenal gland (Figure 3). These lesions were interpreted as metastases of thyroid cancer. At this moment, serum Tg was 369 ng/mL. Due to headaches and confusional state, head CT was performed and showed a frontal lytic lesion of the skull without endocranial involvement. Pain was managed with opioid treatment, and the patient was considered for palliative care. Eventually, clinical deterioration occurred and the patient died a few weeks later.

## 3. Discussion

The evaluation of a mediastinal mass is challenging, and obtaining a prompt histological examination is crucial to ensure an early diagnosis and treatment. Thyroid carcinoma can rarely present as an intrathoracic mass, with few cases described in the literature [1–3]. In such situations, it is difficult to differentiate between metastatic occult thyroid carcinoma and malignant transformation of ectopic thyroid tissue [2, 4]. Surgical excision of the malignant lesion is the treatment of choice. Total thyroidectomy is essential to differentiate between ectopic and malignant thyroid carcinoma, but it should be performed based on individualized risk stratification [4].

Our patient presented with an intrathoracic mass with clear demarcation from the adjacent thyroid gland and pulmonary metastases. Histological examination of the mediastinal mass revealed PTC. In this case, distinction between primary intrathoracic goiter based on an ectopic thyroid or a secondary goiter was not possible because the patient did not have access to surgery and autopsy was not performed. The two largest thyroid nodules found through neck US had a spongiform appearance, and the cytologic result was benign. Bilateral micronodules in the thyroid gland were not biopsied. Although it is rare, a few cases of microcarcinomas with metastases have already been reported [5].

Secondly, the histopathological classification of the tumor revealed a differentiated thyroid carcinoma, with positivity of PAX8 and TTF1. The histological images were suggestive of PTC showing papillary structures and nuclear pseudoinclusions. However, this diagnosis has to be interpreted with caution, since the demonstration of PTC requires multiple histological images picked up at different locations, all showing the features of PTC. A biopsy was not obtained due to the location of the tumor and the patient's general condition.

In this case, there was rapid disease progression with PET-FDG showing multiple lesions with intense uptake, regarded as a malignant, less-differentiated disease. This clinical course is rare for highly differentiated PTC, but it seems to be more frequent as an undifferentiated transformation of PTC or as a poorly differentiated thyroid carcinoma. However, PET-FDG was not performed at diagnosis because diagnostic evaluation was initially conducted in another institution. PET-FDG imaging is a useful tool in poorly differentiated thyroid carcinomas in initial staging and in the follow-up to evaluate metastatic disease. In the authors' view, the tumor was probably heterogenous with mixed areas, and the cytological analysis identified the differentiated component only. Tumors based on an undifferentiated transformation of PTC are often observed to have a mixture of undifferentiated and differentiated sites [6]. Nevertheless, the involvement of the myocardium, muscle, and adrenal by thyroid carcinoma is rare, and only few cases have been reported in the literature [7, 8].

Thyroid carcinoma presenting with concurrent hyperthyroidism due to Graves' disease is also uncommon [9, 10]. ^99m^Pertechnetate scintigraphy showed diffuse uptake in the thyroid parenchyma but no uptake in the paratracheal mass. One hypothesis is that the increased uptake in the hyperfunctioning thyroid gland probably suppressed the uptake in the mediastinal mass, similar to toxic adenoma or struma ovarii [11]. Another explanation is the dedifferentiation of the mediastinal and lung metastases with subsequent loss of sodium iodide symporter which is needed for pertechnetate to enter the cells.

The authors chose to report this case despite the unfavorable outcome because of its unique and unexpected presentation and symptoms. Thyroid cancer, although rare, is a possible diagnosis to consider in the presence of a mediastinal mass.
